# Infection of expanded polytetrafluoroethylene and Dacron-coated stents with *Staphylococcus epidermidis*: an experimental study in pigs

**DOI:** 10.1590/1677-5449.200157

**Published:** 2021-06-25

**Authors:** Clandio de Freitas Dutra, Adamastor Humberto Pereira, Claudia Wollheim, Rodrigo Pongiluppi, Roberto Fellini, Sérgio Ventura Gomes, Henrique Nonemacher

**Affiliations:** 1 Universidade de Caxias do Sul – UCS, Caxias do Sul, RS, Brasil.; 2 Universidade Federal do Rio Grande do Sul – UFRGS, Porto Alegre, RS, Brasil.; 3 Hospital Santa Mônica Erechim, Erechim, RS, Brasil.

**Keywords:** stent, infection, Staphylococcus epidermidis, aorta, pigs, stent, infecção, Staphylococcus epidermidis, aorta, porcos

## Abstract

**Background:**

Diagnosis of the etiologic agent of endoprosthesis infections is essential to enable treatment, since these infections constitute important complications of endovascular procedures. Sonication of explanted tissue and materials is a technique that can be used to facilitate detection of biofilm-producing bacteria.

**Objectives:**

To evaluate infection of pigs' aortas after implantation of nitinol stents coated with polytetrafluoroethylene (ePTFE) or Dacron, previously infected with biofilm-producing *Staphylococcus epidermidis*. Intimal thickening and the inflammatory response in the aortic wall were also evaluated.

**Methods:**

11 ePTFE-coated nitinol stents and 10 Dacron stents infected with *S. epidermidis* strains were implanted in the infrarenal aorta of 21 8-week-old pigs. After 2 weeks, the aorta containing the stents was removed. A vortex mixer and ultrasound were used to homogenize the samples and remove the biofilm. Subsequently, the number of colony-forming units was counted.

**Results:**

There were no significant differences between the two groups in terms of the number of colony-forming units or of inflammation in the arterial wall. With the exception of one specimen from the Dacron group, all aortic stent cultures were positive for *S. epidermidis*.

**Conclusions:**

There were no significant differences in the inflammatory response or infection rate between ePTFE and Dacron-coated stents actively infected with biofilm-producing *S. epidermidis*. Intimal thickening and the inflammatory response to infection of endoprostheses were similar. These results suggest that the two most widely used stent lining materials have a similar infection rate.

## Resumo

**Contexto:**

O diagnóstico do agente etiológico é essencial para o tratamento das infecções de endoprótese, pois representam uma importante complicação do tratamento endovascular. A sonificação dos tecidos pode ser uma técnica usada para auxiliar na detecção de bactérias produtoras de biofilme.

**Objetivos:**

Avaliar a infecção da aorta dos porcos após o implante de stents de nitinol revestidos com politetrafluoretileno (ePTFE) ou Dacron, infectados com *Staphylococcus epidermidis*, produtor de biofilme. O espessamento intimal e a resposta inflamatória na parede aórtica também foram avaliados.

**Métodos:**

Onze stents de nitinol revestidos com ePTFE e 10 stents de Dacron infectados com cepas de *S. epidermidis* foram implantados na aorta infrarrenal de 21 porcos com 8 semanas de idade. Após duas semanas, a aorta contendo os stents foi removida. Um misturador de vórtice e ultrassom foram utilizados para homogeneizar as amostras e remover o biofilme. Posteriormente, o número de unidades formadoras de colônias foi contado.

**Resultados:**

Não houve diferenças significativas no número de unidades formadoras de colônias ou inflamação na parede arterial entre os dois grupos. Todas as culturas de stent aórtico foram positivas para *S. epidermidis*, exceto uma no grupo Dacron.

**Conclusões:**

Não houve diferenças significativas na resposta inflamatória ou na taxa de infecção entre os stents revestidos de ePTFE e Dacron, infectados ativamente pelo *S. epidermidis* produtor de biofilme. O espessamento intimal e a resposta inflamatória à infecção das endopróteses foram semelhantes. Esses resultados sugerem que os dois materiais de revestimento de stent mais amplamente utilizados têm uma taxa de infecção semelhante.

## INTRODUCTION

*Staphylococcus epidermidis (S. epidermidis)* is one of the most common pathogens in infections of devices implanted in the vascular system.^1^^-^3 Diagnosis of endoprosthesis infection is usually made on the basis of clinical findings, imaging studies, and microbiological tests. It is yet to be determined whether there are differences in infection rates between ePTFE and Dacron, two materials used to coat stent grafts and endoprostheses, or their influence in clinical practice.^4^

Furthermore, there has been a considerable increase in the number of reports of infections in stents and endoprostheses, which is due to their increasing use worldwide, with incidence rates of 0.3 to 3%, and when surgery is performed to explant endoprostheses in aortic locations, operative mortality can reach 30%.^5^^-^9

The pathogenesis of stent graft infection is multifactorial. Early infections are generally caused by failures of sterility during implantation or by presence of bacteria in the aneurysmal thrombus. Later infections are mainly caused by the hematogenous spread of bacteremia (usually originating in the urinary or respiratory tracts), by bacterial translocation, or by iatrogenic contamination during the surgical procedure.^4^^,^^7^

It is extremely important to identify the microorganism causing the infection, since the patient can then be provided with the best treatment. Using the different sampling techniques available, microorganisms can be isolated in about 75% and 98% of cases (4). Accurate diagnosis of the etiologic agent causing the infection is not always possible, because some strains of *S. epidermidis* produce biofilm and have the ability to adhere to and colonize synthetic materials, which makes it difficult to isolate the bacteria in conventional cultures.^1^^,^^10^^,^^11^ Sonication of the explanted material is characterized as an important method for diagnosis of the etiologic agent.^12^^-^14

This study evaluated the number of colony forming units (CFU), intimal and total wall thickening, and the degree of inflammatory response in the two most common types of coated stents (ePTFE or Dacron) previously contaminated with *S. epidermidis* and implanted in pigs’ aortas.

## MATERIAL AND METHODS

A pilot study was carried out with 10 pigs in order to analyze infections in the aorta after implantation of stents coated with Dacron or ePTFE previously infected with *S. epidermidis*. A biofilm-producing strain of *S. epidermidis* bacteria was selected from among 16 others that infected central venous catheters in humans.

In addition, were implanted stents coated with Dacron or ePTFE measuring 8 mm in diameter and 25 mm in length (Laboratory of Mechanical Manufacturing, School of Engineering at the Federal University of Rio Grande do Sul) in the pigs' aortas. After 14 days, the pigs' aortas were explanted with the coated and previously infected stents. The stents were sent for conventional anatomopathological examinations and culturing.

In the main study, samples of coagulase-negative *Staphylococcus* were isolated from the tips of 16 infected venous catheters from hospitalized patients. Biofilm production was evaluated using the conventional methods described by Heilmann et al.^15^ and by macroscopic observation of the biofilm on the surface of a glass sheet, ELISA plate (plastic), and segments of Dacron and ePTFE grafts, as described by Cucarella et al.^16^

The material containing the bacteria was placed in 3 mL of triptych soy broth (TSB, Merck®) supplemented with 1.0% glucose and incubated for 18 hours at 35 ± 1 °C. Thereafter, it was diluted in 50 mL of supplemented TSB at a concentration of 10^4^ to 10^5^ CFU/mL measured in a counting chamber. Both types of stents were immersed and incubated for 1 h at 35 ± 1 °C. The stents were removed, washed with sterile saline and maintained in test tubes with 0.9% saline until implantation.

Blood samples were collected before and after surgery to analyze erythrocyte sedimentation rate (ESR), C-reactive protein (CRP), blood cell counts, hemoglobin, hematocrit, and platelet counts. The animals were weighed daily and rectal temperatures were measured twice a day.

A total of 21 female pigs weighing 18.5 to 19.6 kg were submitted to implantation of stents coated with ePTFE (11 animals) or Dacron (10 animals). After induction of anesthesia, tracheal intubation, and general anesthesia, an extra-peritoneal approach was used to access the aorta under strict sterile conditions. The terminal aorta, iliac arteries, and median sacral arteries were exposed and repaired with vessel loops.^17^ The stents were loaded into a 12F sheath and introduced into the terminal aorta through an arteriotomy. After stent release in the infrarenal aorta, the arteriotomy was closed with a 6-0 polypropylene suture and the presence of bleeding was verified. No antibiotics were administered in the perioperative period.

On the 14th day, all animals were subjected to median laparotomy under general anesthesia and sterile conditions. The aorta and stent were removed and patency was checked macroscopically. The retroperitoneum was inspected to determine any changes secondary to the infection. The proximal and distal borders of the aorta that contained the stent were identified and properly marked, placed in 10% formalin and sent to the laboratory for histopathological examination. After removal of the aorta containing the stent, the animals were euthanized.

The samples were then sent to the laboratory, placed in 2 mL of sterile saline, homogenized by vortexing for 30 seconds, sonicated (Ultra Cleaner at 41 kHz - Unique®) for 5 minutes, and then homogenized again for another 30 seconds, as described by Trampuz et al.^12^

Dilutions of 1:10, 1:100, and 1:1000 were used for qualitative cultures, and 100 mL of the solution was seeded on blood agar (BioMérieux®) and incubated for 48 hours at 35 ± 1 °C in microaerophilic atmosphere for later identification and colony counting. Cultures were classified as positive when more than one CFU was found.

Additionally, during histopathological examination, intimal and total wall thickening were measured and the aortic wall was examined for presence of bacteria and inflammatory cells.

Student's *t* test was used for statistical analysis of all paired samples and quantitative analysis of CFU counts, and Fisher's exact test was used for analysis of histopathological results.

This study was evaluated and approved by the Graduate and Research Group at the Hospital de Clínicas of the Federal University of Rio Grande do Sul (UFRGS), Porto Alegre, Brazil, and by the Center for Basic Sciences and Health at the University of Caxias do Sul (UCS), Caxias do Sul, Brazil.

The experimental study was carried out at the Microbiology and Pathology Laboratory at the Universidade de Caxias do Sul (UCS) and the UCS Animal Laboratory. Microbiological and laboratory tests were performed at the Fleming Laboratory, Caxias do Sul, Brazil.

## RESULTS

In the pilot study, positive cultures were not obtained for any of the 10 animals, as only conventional culture methods were used and sonication of the explanted tissues and materials from the pigs' aorta was not performed.

In the main study, 19 animals were analyzed: 10 with ePTFE-coated stents and 9 with Dacron-coated stents. Macroscopic analyses showed that all stents were patent at the end of the experiment. Enlarged retroperitoneal lymph nodes were seen in only one animal, in the ePTFE group. Two pigs died: one with an ePTFE-coated stent due to acute stent thrombosis, and the other, with a Dacron®-coated stent, due to aortic perforation.

Mean weight, temperature, operating time, and standard deviations are shown in Tables 1 and 2. There were no statistical differences between the two groups.

**Table 1:** Weight, preoperative temperature, operating time, erythrocyte sedimentation rate, and C-reactive protein.

**Variable**	**ePTFE (n = 10)**		**Dacron® (n = 9)**		**P**
	**Mean**	**SD**	**Mean**	**SD**	
Weight (kg)	18.50	2.12	19.65	3.17	0.37
Preoperative temperature (°C)	37.83	1.08	37.66	1.74	0.81
Operating time	44.00	8.43	46.11	13.64	0.68
ESR	13.00	15.80	12.17	8.32	0.91
CRP	9.33	6.08	9.75	6.36	0.89

SD: Standard Deviation; ESR: erythrocyte sedimentation rate; CRP: C-reactive protein. Mean values were not significant (p > 0.05; Student’s t test).

**Table 2:** Preoperative and postoperative red blood cell, white blood cell, and platelet counts.

**Variables**	**PRE**		**Dacron® (n = 8)**		**POST**		**Dacron® (n = 8)**		**p1**	**p2**
	**ePTFE (n = 9)**				**ePTFE (n = 9)**					
	**Mean**	**SD**	**Mean**	**SD**	**Mean**	**SD**	**Mean**	**SD**		
RBC (million)	5.59	0.60	5.83	0.37	5.90	0.72	5.84	1.17	0.58	0.60
Hemoglobin	9.78	1.04	10.16	0.71	9.88	1.55	10.13	1.78	0.94	0.89
Hematocrit (%)	33.76	5.03	34.45	3.49	33.44	4.98	33.48	5.79	0.72	0.86
Leukocytes	13513	5087	14933	6858	13300	5119	10850	2882	0.25	0.30
Eosinophils (%)	1.63	1.40	2.17	1.47	1.50	0.92	1.00	0.63	0.15	0.24
Band forms (%)	2.13	1.55	3.00	2.00	2.38	2.87	1.83	0.98	0.62	0.44
Granulocytes (%)	45.25	13.49	44.67	15.71	40.50	18.10	45.67	18.73	0.76	0.64
Lymphocytes (%)	47.75	14.73	46.83	14.93	52.13	19.47	48.33	19.22	0.66	0.83
Monocytes (%)	3.25	3.01	3.33	2.58	3.50	1.19	3.00	1.26	0.97	0.77
Platelets	477200	74380	427500	42530	528600	95370	485500	267470	0.41	0.96

SD: Standard Deviation; RBC: red blood cells. Mean values were not significant (p > 0.05; Student’s t test). p1 = time; p2 = interaction time X stent.

Values for blood count, hematocrit, hemoglobin, platelets, and white blood cell count were similar to preoperative results (Table 3). Weight, ESR, and CRP were higher after surgery, but differences between groups were not significant (Table 4).

**Table 3:** Preoperative red blood cell, white blood cell, and platelet counts.

**Variable**	**ePTFE (n = 10)**		**Dacron® (n = 9)**		**P**
	**Mean**	**SD**	**Mean**	**Standard**	
RBC (million)	5.59	0.60	5.83	0.37	0.41
Hemoglobin (g %)	9.78	1.04	10.16	0.71	0.46
Hematocrit (%)	33.76	5.03	34.45	3.59	0.78
Leukocytes	13512	5087	13933	6858	0.66
Eosinophils (%)	1.63	1.40	2.17	1.47	0.50
Band forms (%)	2.13	1.55	3.00	2.00	0.37
Granulocytes (%)	45.25	13.49	44.67	15.71	0.94
Lymphocytes (%)	47.75	14.73	46.83	14.93	0.91
Monocytes (%)	3.25	3.01	3.33	2.58	0.96
Platelets	477200	74380	427500	42530	0.28

SD: Standard Deviation; RBC: red blood cells. Mean values were not significant (p > 0.05; Student’s t test).

**Table 4:** Preoperative and postoperative weight, erythrocyte sedimentation rate, and C-reactive protein.

**Variables**	**PRE**				**POST**				**p1**	**p2**
	**ePTFE (n = 9)**		**Dacron® (n = 8)**		**ePTFE (n = 9)**		**Dacron® (n = 8)**			
	**Mean**	**SD**	**Mean**	**SD**	**Mean**	**SD**	**Mean**	**SD**		
Weight (kg)	18.81	2.04	20.11	3.05	24.51	2.97	23.28	6.21	< 0.001	0.23
ESR	13.00	15.80	12.17	8.32	15.88	21.45	5.67	3.83	0.69	0.31
CRP	9.33	6.08	9.75	36	17.33	17.60	20.50	29.54	0.12	0.81

SD: Standard Deviation; ESR: erythrocyte sedimentation rate; CRP: C-reactive protein. Mean values were not significant (p > 0.05; Student’s t test). p1 = time; p2 = interaction time X stent.

Mean body temperature of the pigs was 37.8 °C in the ePTFE group and 37.6 °C in the Dacron group. Eight of the nine (89%) samples of Dacron®-coated aortic stents and all ePTFE-coated stents were positive for *S. epidermidis*. There were no statistically significant differences in number of CFU between the two groups (Table 5) (p = 0.434).

**Table 5:** Quantitative results of cultures of *S. epidermidis* (CFU/mL) in aortic stent samples.

**Coating**	***Staphylococcus epidermidis* (CFU/mL)**			**p**
	**Min**	**Max**	**Mean**	
ePTFE (n = 9)	2.5 X 10^5^	1.5 X 10^6^	7.9 X 10^5^	0.434
Dacron® (n = 8)	0.0	2.2 X 10^6^	1.5 X 10^5^	

CFU: colony-forming units. Mean values were not significant (p > 0.05; Student t test).

In the histopathological analysis, the proximal and distal edges of the aorta were compared with normal aorta distant from the area of stent deployment. Aortic samples from animals with ePTFE-coated stents had greater increases in intimal thickness and total wall thickness and more inflammatory cells than the group with Dacron®-coated stents. The results of the histopathological analysis are shown in Table 6. No bacteria were found in any of the samples during histopathological analyses.

**Table 6:** Histopathological analysis of the proximal and distal edges of the aorta after implantation of Dacron®- or ePTFE-coated stents.

**Variables**	**Proximal edge**		**p**	**Distal edge**		**p**
	**ePTFE (n=9)**	**Dacron® (n =8)**		**ePTFE (n=9)**	**Dacron® (n=8)**	
Intimal thickening (%)	33.0	0.0	0.206	44.4	37.5	>0.999
Wall thickening (%)	11.1	0.0	>0.999	0.0	25.0	0.206
Inflammation (%)	33.0	0.0	0.206	44.4	25.0	0.62
Colony	0.0	0.0	0.0	0.0	0.0	0.0

Mean values were not significant (p > 0.05) Fischer’s exact test.

## DISCUSSION

Infection of endovascular devices is an uncommon complication, but is extremely serious and difficult to treat. When suspected, endoprosthesis infections should be promptly investigated and treated aggressively.^9^^,^^18^^-^20 It is therefore extremely important to identify the etiologic agents causing infections, since they are directly related to increased patient morbidity and mortality.^4^^,^^10^^,^^13^

Currently, most endovascular devices are covered by one of two different polymers, Dacron® or ePTFE. The choice of these polymers and the metallic endoprosthesis skeleton is mainly related to their chemical and mechanical stability, rather than properties that inhibit colonization by microorganisms.^4^ There is still no consensus on which of these materials best resists infection.^21^ In our study, we obtained similar infection rates in both coated stents.

Blood cultures were obtained from all animals before the operation, after the operation, and before removal of surgical samples, and all were negative. Stents were implanted through an arteriotomy to minimize exposure and contamination of the device.^17^ Additionally, the animals' temperatures varied daily (Table 1), but none of them had fever. Average temperatures were 37.8 °C in the ePTFE group and 37.6 °C in the Dacron group, indicating that bacterial virulence was not elevated, since temperatures of up to 42 °C are normal in pigs.^22^

In general, stent infections involve progressive arterial destruction, stent thrombosis, septic embolization, and pseudoaneurysm formation.^23^ Acute ePTFE-coated stent thrombosis occurred in one of the pigs. In another, with a stent coated with Dacron®, there was perforation of the aorta. Both animals died during the operation. We believe that these deaths were not related to endoprosthesis infection.

Microscopic examination revealed signs of inflammation in some samples of the aortic wall, with macrophages, plasma cells, and lymphocytes, but there was no predominance of neutrophils (acute inflammation cells), as found in studies by Thibodeaux et al.^24^ and Hearn et al.,^25^ who infected stents with *S. aureus*.

Different culture techniques have been used for diagnosis of infection in vascular prostheses.^10^^,^^21^^,^^26^ Cultures may be negative because of use of antibiotics or presence of biofilm-producing microorganisms that adhere strongly to synthetic materials. There can also be false positives caused by contamination during endoprosthesis removal.^25^^,^^26^ Moreover, vascular prosthesis infections can be under-recognized when identified by standard culture techniques.

Sonication of the graft material with subsequent culture and broad-spectrum PCR was proposed by Ulcar et al.^13^ and Waldvogel^14^ to improve bacterial detection. Thus, graft sonication and wide-spectrum PCR of the sonified fluid can contribute to optimized antimicrobial treatment, since they achieved a bacterial recovery rate of 31.8% with standard culture, 66.7% with PCR, and 72.2% in sonified tissues.^13^

Trampuz et al.^12^ used sonication to dislodge biofilm and found positive culture rates of up to 78.5% in cultures of synovial fluid from knees and hips. Bergamini et al.^10^ showed that rates of bacterial recovery from Dacron® grafts contaminated with biofilm-producing *S. epidermidis* were 30% using agar alone, 72% using broth culture medium, and 83% using broth culture medium and ultrasound. In our pilot study, in which tissue sonication was not used, we were unable to isolate the strains of *S. epidermidis* previously inoculated. In comparison, in our main study we obtained 100% bacterial isolation from ePTFE-coated stents and 89% from those coated with Dacron®, which indicates that ultrasound is an effective method for diagnosing the etiologic agent causing infections.

## CONCLUSIONS

Identification of the microorganism causing an infection is extremely important in order to provide the best treatment for the patient. Our results indicate that ultrasound should be used routinely by microbiologists to more effectively detect the presence of biofilm-producing bacteria, since the recovery rate of *S. epidermidis* bacteria from infected stents was high when ultrasound was used.

Nitinol stents coated with ePTFE or Dacron® implanted in the aortas of pigs had similar results for number of colony forming units and degree of wall inflammation when infected with *S. epidermidis*. We believe that the coating material does not significantly influence infection rate and most stent infections are mainly due to introduction of bacteria at the time of implantation.
